# Fathers today: design of a randomized controlled trial examining the role of oxytocin and vasopressin in behavioral and neural responses to infant signals

**DOI:** 10.1186/s40359-019-0356-2

**Published:** 2019-12-16

**Authors:** Annemieke M. Witte, Marleen H. M. de Moor, Marinus H. van IJzendoorn, Marian J. Bakermans-Kranenburg

**Affiliations:** 1 Clinical Child & Family Studies, Faculty of Behavioral and Movement Sciences, Vrije Universiteit, Amsterdam, Van der Boechorststraat 7, 1081 BT The Netherlands; 2 Department of Psychology, Education, and Child Studies, Erasmus University Rotterdam, Rotterdam, Burg. Oudlaan 50, 3062 PA The Netherlands; 3 Leiden Institute for Brain and Cognition, Leiden University Medical Center, Leiden, 2300 RC The Netherlands

**Keywords:** Fathers, Oxytocin, Vasopressin, Parenting, fMRI

## Abstract

**Background:**

Previous research has mostly focused on the hormonal, behavioral and neural correlates of maternal caregiving. We present a randomized, double-blind, placebo-controlled within-subject design to examine the effects of intranasal administration of oxytocin and vasopressin on parenting behavior and the neural and behavioral responses to infant cry sounds and infant threat. In addition, we will test whether effects of oxytocin and vasopressin administration are moderated by fathers’ early childhood experiences.

**Methods:**

Fifty-five first-time fathers of a child between two and seven months old will participate in three experimental sessions with intervening periods of one to two weeks. Participants self-administer oxytocin, vasopressin or a placebo. Infant-father interactions and protective parenting responses are observed during play. Functional Magnetic Resonance Imaging (fMRI) is used to examine the neural processing of infant cry sounds and infant threat. A handgrip dynamometer is used to measure use of handgrip force when listening to infant cry sounds. Participants report on their childhood experiences of parental love-withdrawal and abuse and neglect.

**Discussion:**

The results of this study will provide important insights into the hormonal, behavioral and neural correlates of fathers’ parenting behavior during the early phase of fatherhood.

**Trial registration:**

Dutch Trial Register: NTR (ID: NL8124); Date registered: October 30, 2019.

## Background

Parenting behavior in non-human mammals is influenced by endocrine systems [1]. Hormonal processes are also implicated in human mothering and fathering behaviors [2]. Several correlational studies in humans have shown associations between oxytocin and vasopressin levels and parent-child interactions [3–5]. Moreover, experimental studies with intranasal administration of oxytocin and vasopressin have shown effects on human parenting behavior and neural responses to infant signals [6–11]. Whereas most previous research has focused on the hormonal, behavioral and neural correlates of maternal caregiving, the present study will examine the hormonal, behavioral, and neural dynamics of paternal behavior in first-time fathers during a specific phase of fatherhood: between 2 and 7 months after the baby has been born. In this protocol, we present a randomized, double-blind, placebo-controlled within-subject trial to examine the effects of intranasal administration of oxytocin and vasopressin on parenting behavior and the neural and behavioral responses to infant signals. In addition, we will examine whether effects of oxytocin and vasopressin are moderated by fathers’ early childhood experiences.

### Oxytocin and vasopressin in infant-father interactions

Oxytocin and vasopressin are key hormones involved in social and affiliative processes, including human parenting behaviors [2]. Oxytocin and vasopressin have been associated with the expression of paternal behavior [3–5]. In the first months of parenthood, oxytocin levels appear to be similar in mothers and fathers, although they seem associated with different interaction styles. Infant-mother interactions characterized by high levels of affectionate touch were associated with an increase in oxytocin levels in mothers, whereas infant-father interactions characterized by high levels of stimulatory contact were associated with an increase in oxytocin levels in fathers [5]. Experimental studies showed that fathers with typically developing children and fathers of children with autism spectrum disorder were more stimulating of their child’s exploration and autonomy, and showed less hostility after receiving intranasal oxytocin administration compared to the placebo condition [7, 8].

Finally, experimental evidence showed that administration of vasopressin increased expectant fathers’ interest in direct care for children compared to the control group [6]. In addition, in a large sample of 119 fathers with 4 to 6-month-old infants, higher vasopressin levels were correlated with more stimulatory contact [3].

### Oxytocin and vasopressin in paternal responses to infant signals

Research has further shown that oxytocin and vasopressin affect neural and behavior responses to infant signals [4, 9, 10, 12–14]. For example, a small functional magnetic resonance imaging (fMRI) study including fathers of 4- to 6-month-old infants reported a negative association between fathers’ endogenous vasopressin levels and activations in the inferior gyrus and insula when watching their own infant play, suggesting that lower vasopressin levels enhances social-cognitive and empathic responses, although replication of these findings in larger samples is needed [4].

However, most research examining the effects of oxytocin and vasopressin on neural and behavior responses to infant signals has been conducted in samples of women or mothers. For example, a double-blind experimental study including a sample of 42 nulliparous women showed that they used less excessive handgrip force when listening to infant cry sounds after receiving intranasal oxytocin administration compared to women in the control group, but this result was only found for women who had no or few childhood experiences of harsh discipline [13], and for nulliparous women with insecure attachment representations [10]. Reduced handgrip force after oxytocin administration may represent a more sensitive caregiving response to a crying infant, although this response seems to be dependent on individual characteristics and experiences. A speculative explanation for this dependency may be that individual characteristics and experiences result in epigenetic changes at the oxytonergic receptor level, which may in turn lead to decreased sensitivity for the effects of intranasal administration of oxytocin [15].

On a neural level and in the same sample of Bakermans et al. [13], nulliparous women in the oxytocin condition showed less neural activation in the amygdala and increased neural activation in the insula and inferior frontal gyrus when listening to infant cry sounds as compared to women in the placebo condition [9]. This pattern of neural activation may suggest that oxytocin reduces anxiety and aversion and enhances empathic understanding towards the distressed infant [9].

Interestingly, an fMRI study in a sample of 15 fathers of children aged between 1 and 2 years reported that oxytocin administration did not reduce neural activation in the amygdala nor affected activation in other brain areas when fathers listened to infant cry sounds [14]. Oxytocin administration enhanced neural responses when father’s viewed pictures of their own children in the caudate nucleus, dorsal anterior cingulate and visual cortex, suggesting enhanced activation in the reward-related circuities of the brain when fathers view pictures of their own child [14]. Although findings of Li et al. [14], were based on a small sample and further investigation with a larger sample is needed, it should also be noted that differences in neural activation in response to infant cry sounds may emerge as a result of sex-specific neural adaptations following parenthood [16].

In contrast to oxytocin administration, it is less well known how vasopressin administration affects behavioral responses to infant signals and whether effects are dependent on individual characteristics and experiences. A study using a within-subject design showed that vasopressin administration enhanced the use of excessive force in a sample of 25 expectant fathers while viewing a picture of an unfamiliar infant compared to viewing a morphed picture of the expectant father’s own infant, while reversed results were found in the placebo condition [12]. It was suggested that vasopressin administration may enhance the recognition of related offspring, affecting expectant fathers’ behavioral responses. The use of increased handgrip force to an unknown infant (versus own infant) might be explained by enhanced protective parenting in favor of the own child. No significant correlations were found between expectant fathers’ average handgrip force and expectant fathers’ experiences of caregiving during their childhood. The study did not examine whether expectant fathers’ caregiving experiences during childhood moderated the relation between vasopressin administration and use of handgrip force [12].

The effects of vasopressin administration on neural responses to infant cry stimuli have also been examined. In the same sample of Alyousefi-van Dijk et al. [12], intranasal vasopressin administration increased neural activation in response to infant cry sounds in the anterior cingulate cortex, paracingulate gyrus, and supplemental motor area, suggesting increased empathy and motivation to terminate the infants’ crying [11]. This effect was stronger in expectant fathers who experienced lower levels of parental love-withdrawal during their childhood. Parental love-withdrawal is described as a disciplinary strategy in which the parent withholds love and affection when the child misbehaves or fails at a task [17]. However, in another study it was found that vasopressin administration did not affect the neural processing of infant cry stimuli in fathers of 1–2-year-old children [14].

A meta-analysis including 350 participants from 14 studies [18], largely confirmed and also extended findings of previous research describing the neural circuits involved in infant cry perception [19, 20]. Results of the meta-analysis showed that the auditory system, the thalamocingulate circuit, the dorsal anterior insula, the pre-supplementary motor area and dorsomedial prefrontal cortex, the inferior frontal gyrus and structures related to motoric processing were involved in infant cry perception. Neural activation in response to infant crying was moderated by parenthood such that parents showed more activation in the bilateral auditory cortex, posterior insula, pre- and postcentral gyrus and right putamen compared to non-parents, while non-parents showed more neural activation in the right caudate nucleus than parents [18].

### Oxytocin and vasopressin in protective parenting

Finally, oxytocin and vasopressin levels may be related to protective parenting behaviors. Animal studies have shown evidence for the involvement of hormonal process in the protection of offspring. For example, oxytocin release in the brain of rats was positively associated with maternal offence attacks towards an intruder placed into the cage [21]. Moreover, administration of synthetic oxytocin and infusion of an oxytocin receptor antagonist resulted in, respectively, an increase and decrease in maternal aggression towards a cage intruder. Finally, binding of oxytocin to receptors in the lateral septum was positively related with a peak in maternal aggressive behaviors in rats [22]. In monogamous male prairie voles, vasopressin injections increased and vasopressin antagonists terminated aggressive territorial behaviors towards intruders [23]. This increase in territorial protection, affected by higher vasopressin levels, supports the provision of a safe environment and facilitation of partner and offspring protection. These results show that in non-human mammals oxytocin and vasopressin are involved in the expression of aggressive behaviors in situations of threat, which is in line with results of experimental studies conducted in humans.

In a double-blind between-subject design in which men self-administered oxytocin or a placebo, oxytocin increased in-group trust and cooperation but at the same time increased defense aggression toward competing out-groups [24]. In another double-blind between-subject study in men, oxytocin administration intensified the emotional modulation of aversive social stimuli in comparison to placebo [25]. Furthermore, a study on the relation between oxytocin administration and protective parenting, conducted in 16 mothers with depression, showed that after oxytocin administration, mothers with a depression showed increased physically and verbally protective behaviors when confronted with a socially intrusive stranger [26].

Research on vasopressin administration in humans implicates a link between vasopressin and male aggressive behavior. In a double-blind between-subject study, administration of vasopressin in healthy men enhanced electromyography activity of the left corrugator supercilii in response to viewing neutral facial expressions, resulting in similar magnitudes of activation when viewing angry and neutral facial expressions [27]. It was speculated that administration of vasopressin may stimulate aggressive behaviors in males by biasing them to respond to neutral facial stimuli as if they were threatening.

The neural underpinnings of paternal protection have received little attention. An fMRI study (with no focus on hormonal effects) in 21 fathers explored pre- and postnatal neural activation in response to viewing infants in situations of threat and reported increased brain activation for infant threatening versus neutral situations in the amygdala and various cortical and subcortical regions in pre- and postnatal fatherhood [28]. The amygdala has been consistently associated with the detection of salience and threat [29, 30], and may play a pivotal role in protective parenting behavior. Results further indicated that neural responses to infant threat were associated with protective paternal behavior in everyday life [28]. These findings are the basis for a better understanding of the neural correlates of protective paternal behavior. It has not yet been examined how oxytocin and vasopressin administration affect the neural processing of threat to the infant.

### Current study

In order to shed further light on the underlying mechanisms of fathers’ parenting behavior, the present study focuses on the hormonal, behavioral and neural underpinnings of fatherhood. In the current study, we will examine the effects of oxytocin and vasopressin administration on parenting behavior and the neural and behavioral responses to infant signals. We use a randomized double-blind within-subject design and focus on first-time fathers in the early postnatal period with a baby is between 2 and 7 months old.

### Aims and hypotheses

Our first aim is to examine how intranasal administration of oxytocin and vasopressin affects fathers’ behavioral responses to infant signals. Our second aim is to examine how intranasal administration of oxytocin and vasopressin affects neural responses to infant cry sounds and infant threat. Our third aim is to explore brain-behavior associations taking into account the effects of oxytocin and vasopressin administration. Our final aim is to explore whether effects of oxytocin and vasopressin administration on behavioral and neural responses to infant cry sounds and infant threat are moderated by fathers’ early childhood experiences. We hypothesize that infant-father interactions in the oxytocin and vasopressin condition are characterized by enhanced stimulatory and sensitive play and increased protective paternal behavior as compared to the placebo condition. We further expect that oxytocin and vasopressin administration affect behavioral responses to infant cry sounds and neural responses to infant cry sounds and threat to the infant.

## Methods/design

### Study design

The current study will employ a randomized-double-blind, placebo-controlled within-subject design. Fifty-five first-time fathers of a child aged between two and seven months old will visit our research center for three experimental sessions. The experimental sessions include three conditions: intranasal administration of [1] oxytocin [2], vasopressin, and [3] a placebo. Participants will be randomly assigned to order of administration. Participants and researchers are blind to order of administration. The experimental sessions will take place with intervening periods of one to two weeks. The datasets generated and analyzed in the current study are archived in accordance with the University implementation of the National Guidelines for Archiving of Academic Research. Datasets will be made available from the senior author upon reasonable request .

### Participants

#### Recruitment

Participants will be recruited through social media, folders and municipality records. Municipalities will send fathers of newborn infants on our behalf invitation letters to participate in the study. Fathers can express their interest for participation with an attached response card. Interested fathers will receive a letter with detailed information about the study and inclusion criteria are checked in a phone call. In order to be eligible for participation, participants must meet the following inclusion criteria: first-time fathers with a child between two and seven months old, living in the same house as their partner and baby, both parents must have parental authority. Participants will be excluded from the study in case of a history of or when currently suffering from neurological disorders, endocrine diseases, psychiatric disorders, cardiovascular diseases, use of psychoactive medications, nose injuries and disorders, or magnetic resonance imaging contraindications.

Participants will receive a financial reward that increases in value (to a maximum of €130) for each session completed: €30 after the first session, €40 after the second session, and €50 after the third session. At the final visit, participants will receive a small, age-appropriate gift for the child. Participants will receive an extra €10 after the final visit if they have completed at least 80% of the questionnaires. Travel expenses will be covered. The partner of the father is invited to accompany the father and infant to our research center. In the event the partner is not able to join the visit, we will arrange childcare by an experienced babysitter chosen or approved by the parent. Any childcare costs will be covered.

#### Randomization

**Fig. 1:**
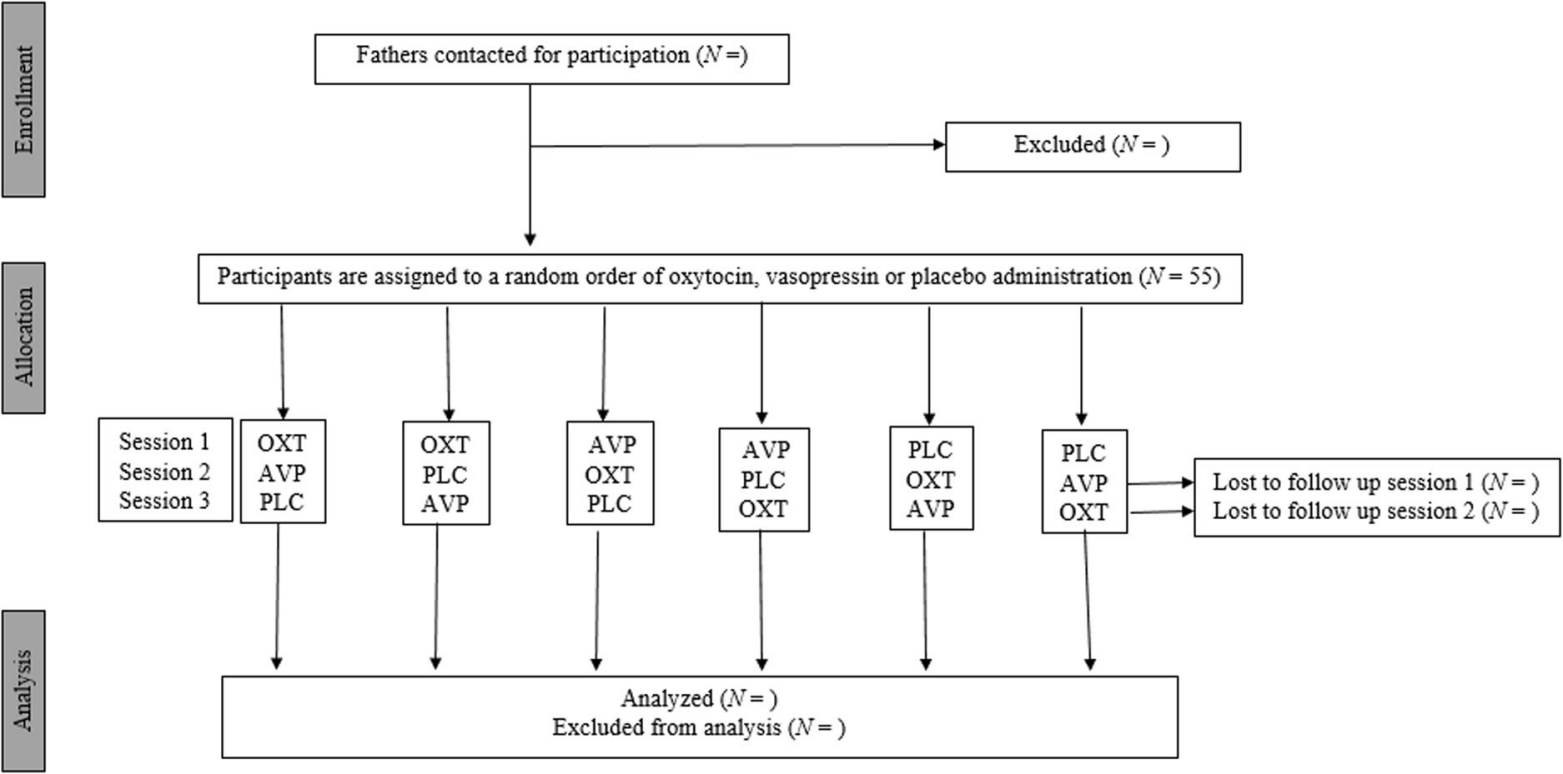
Consort flowchart of the phases of the randomized double-blind placebo-controlled within-subject design. The three conditions imply six possible counterbalanced orders of assignment. All participants are randomly assigned to each of the three conditions (oxytocin, vasopressin, placebo). OXT; Oxytocin, AVP; Vasopressin, PLC; Placebo

Randomization of administration is performed by an independent researcher who is not involved in the study. Randomization is performed before the start of the interventions using a computer-generated randomization sequence. Assigned order of administration is stored in a locked folder in accordance with the University protocol. For a flowchart of the phases of the present randomized control trial, see Fig. 1. At the end of each visit, participants are asked to guess their assignment of condition. After the third visit, participants are provided with the option to be informed about their order of assignment. Participants who want to be informed receive their order of assignment by mail from an independent researcher who is not involved in the study. Researchers are not informed and remain blind to avoid bias that may be generated by knowledge of condition assignment.

#### Sample size and power

In this within-subject experiment, the sample size will be *N* = 55. For vasopressin, the literature on experimental studies with vasopressin administration is scarce, preventing the computation of a pooled effect size via meta-analysis. Based on the literature on oxytocin administration [7, 9, 15, 31], a medium effect size (*f* = 0.25) may be expected but taking into account that some publication bias against studies with small effect sizes may exist, we choose an expected effect size of *f* = 0.20. The program G*Power 3.1.9 estimates the power of specific analyses given an expected effect size and a sample size. For a within-subjects, repeated measures analysis of variance with an expected medium effect size *f* = 0.20, a correlation of the repeated measures *r* = 0.50, an alpha level of 0.05 and age of the child included as a continuous variable affecting the degrees of freedom (df), the power is >.85. The power is > .80 when *N* = 42. Thus, with *N* = 55 we will have sufficient power even if some participants fail to complete the sessions. Similarly, this suggests sufficient statistical power for the effects of vasopressin administration.

### Procedure

In this double-blind placebo-controlled within-subject design, participants are randomly assigned to one of the six counterbalanced orders of conditions. Participants are instructed to self-administer oxytocin (Syntocinon®, 24 IU/ml, registered in the Netherlands as RVG 03716), vasopressin (Vasostrict®, 20 IU/ml), or a placebo (Chlorbutanol solution) using a nasal spray. Self-administration takes place under supervision of a researcher. High doses (> 60 IU) of oxytocin nasal spray may in some cases lead to headache. Based on the single doses of 24 IU/ml used in the present study, side effects are negligible [32, 33]. There are no risks demonstrated to be associated with the administration of vasopressin. All experimental medication is prepared by the hospital pharmacy of the Amsterdam University Medical Centre, the Netherlands. The doses administered are comparable with the doses previously used in research examining the behavioral and neural effects of oxytocin and vasopressin administration [14, 34, 35].

Participants will be instructed to not consume alcohol and abstain from excessive physical activity 24 h before assessments take place and are instructed to not consume any caffeine on the day of assessment. The first behavioral measurement takes place 30 min after intranasal administration of oxytocin, vasopressin or placebo. For an overview of the order of assessments during each session, see Table 1. Each session takes approximately two hours to complete.

**Table 1:** *Order of research assessments during each visit*

1. Hormonal measures - saliva measurement	
2. Intranasal administration of oxytocin, vasopressin or placebo	
3. Hormonal measures – hair assessment ^a^	
4. Questionnaires	
5. Hormonal measures – saliva measurement	
6. Infant-parent interaction and protective parenting ^b^	
7. Neural responses to infant cry sounds	
8. Neural responses to infant threat	
9. Handgrip force in response to infant cry sounds	
10. Hormonal measures - saliva measurement	

*Note*. ^a^ Hair samples are only obtained during the first research visit. ^b^ Protective parenting is only assessed during the second research visit

### Measures

#### Oxytocin and vasopressin measures

A baseline saliva sample is collected prior to administration of oxytocin, vasopressin or placebo, 30 min after administration and at the end of the research visit (approximately two hours after administration). Participants abstain from eating and drinking (only water is approved) during their visit. To measure oxytocin and vasopressin levels, participants chew for 60 s on a cotton swab on which saliva collects. Samples are immediately stored in a refrigerator at − 80 degrees Celsius until laboratory assessment.

#### Infant-parent interaction and protective parenting

Infant-father interactions will be observed during a 10-min play session. Participants are instructed to engage in their usual routines of play. No play material is provided during the first five minutes of the interaction. After 5 min, the researcher will hand the father a bag of toys and the father is instructed that he can use the toys during play. All infant-father interactions are videotaped. Coders will be trained to code interactions for sensitive and stimulatory play. During the second visit, after 10 min of play, fathers and infants are exposed to a short sound fragment (the Auditory Startling Task (AST)) to measure protective paternal behavior. The sound consists of white noise (80-db) for 10 s with short breaks. The sound can elicit a protective paternal response without exposing the infant to any harm. At the end of the sound, the researcher enters the room and apologizes for the sound: “I am sorry; we had some technical problems and it took me a moment to get things under control. Our apologies”. The participant is debriefed about the purpose of the sound fragment at the end of the third session. Protective parenting is only assessed once in order to ensure task reliability. Coders will be trained to code paternal responses for protective parenting behaviors. For all coding material, intercoder reliability (ICC) > .60 will be obtained. Monthly meetings are organized to discuss videos and regular checks will be implemented to prevent coder drift.

#### Neural responses to infant cry sounds

To assess neural responses to infant cry sounds participants listen to cry and scrambled control sounds (adapted from Thijssen et al. [11]). A total of 6 cry sounds are recorded from 6 infants (3 males, 3 females), using a TasCam DR-05 solid-state recorder with at a 44.1 kHz sampling rate and 16 bit. All sounds are recorded between 2 days and 5.5 months after birth. All cry sounds are scaled, the intensity is normalized to the same mean intensity (74 Db) and sounds are edited to last for 10 s using PRAAT software [36]. For each cry sound, a neutral auditory control stimulus is created by calculating the average spectral density over the entire duration of the original sound. A continuous sound of equal duration was re-synthesized from the average spectral density and amplitude modulated by the amplitude envelope, extracted from the original sound. After this procedure, all cry sounds and control sounds are intensity matched. The control sounds are identical to the original auditory stimuli in terms of duration, intensity, spectral content, and amplitude envelope.

A large screen located at the back of the MRI bore, viewable through a mirror mounted on the top of the head coil, is used to display the task. Participants randomly receive one of two pre-programmed semi-random orders. The six infant cry sounds are presented three times (18 trials). The six corresponding control sounds are also presented three times, leading to 36 trials. The task is programmed in E-Prime [37].

Sounds are presented for 10 s while a fixation cross hair remained visible on the screen. Trials are separated by an inter stimulus interval (ISI). To maximize the power of the design, ISI is optimized using a web-based tool called Neurodesign [38]. In each of the two pre-programmed orders, trials are separated by an ISI of variable length ranging from 3.5–8.0 s, with a mean ISI of 4.5 s. Blocks of six trials are separated by rest periods of 15 s. During the ISI and rest periods, a fixation cross hair remains visible. For each sound, the question: “How urgent do you find this sound?” is presented once as white text on a black screen together with the presentation of a Likert answer scale ranging from not urgent to very urgent. Participants use their index finger and ring finger to slide along the answer scale and use their middle finger to answer the question. Questions are self-paced and presented at fixed time points, following the 1th, 2th, 13th, 14th, 25th and 26th trial. All responses are registered using a fiber optic response box (Current Designs, Philadelphia, PA, USA).

#### Neural responses to infant threat

To assess neural responses to infant threat, participants view neutral and threatening videos while imagining that their own infant is shown in the videos (adapted from Van ‘t Veer et al. [28]). Prior to the first visit, participants provide a full-color digital photo of their child with a neutral facial expression. Photographs are edited using Adobe Photoshop CS to remove unwanted background features. Subsequently, images are masked with a black face contour and resized to 640 × 480 pixels. Prior to the start of the task, the picture is shown to familiarize the participant with the edited picture of their child. The edited picture is also used in another task in which we examine use of handgrip force in response to infant cry sounds.

A large screen located at the back of the MRI bore, viewable through a mirror mounted on the top of the head coil, is used to display the task. Videos are separated by an inter stimulus interval (ISI). To maximize the power of the design, ISI was optimized using a web-based tool called Neurodesign [38]. In each of the four pre-programmed semi-random orders, trials are separated by an ISI of variable length ranging from 3.0–8.0 s, with a mean ISI of 4.5 s. Participants receive 1 of the 4 pre-programmed orders of 24 threating and 24 neutral videos. Order is based on study identification number (1 assigned to order 1, 4 to order 4, 5 to order 1, etc.). Each order ensures an equal distribution of 12 neutral and 12 threatening videos during the first half of the task, and 12 neutral and 12 threatening videos during the second half of the task.

Prior to the onset of the task, participants view the edited picture of their own infant together with a written instruction to imagine that their own infant is displayed in the succeeding videos. This instruction together with the edited picture of the infant is shown again after each 8 videos (i.e. 6 times in total). After a brief stimulus interval of 250 milliseconds, the instruction screen advances to one of the four pre-programmed semi-random order of 48 videos with a duration of 6 s each. Neutral and threatening video are displayed twice. Videos are selected out of a pool of twelve threatening videos (e.g. hot tea is accidentally spilled on a baby, a baby stroller accidentally rolls into a river, an adult loses grip of a baby stroller that rolls off a bridge and crashes into a cyclist, a car seat with a baby is accidently pushed down the stairs, a baby accidentally falls off a changing table while being changed, and a car is parked backwards and hits a baby in a car seat which was placed on the parking lot). The threatening videos corresponded with twelve matched neutral videos (e.g. tea is placed on a table next to the baby, a baby stroller does not roll into the river, an adult on top of a bridge safely puts baby stroller on the brakes, a baby lies on the changing table while being changed and a car parks backwards at a safe distance from a baby in a car seat placed on sidewalk). These videos thus contrast situations in which protective action is called for, and situations which requires no protective response. The videos (that will be made available upon request) are filmed using a lifelike baby doll by a professional video production team. A neutral doll represents the baby in the videos. In order to ease the task of imagining their own infant in the videos and to reduce any chance of bias, the depiction of the doll’s face and the faces of the actors is minimized.

#### Handgrip force in response to infant cry sounds

Participants are asked to squeeze a handgrip dynamometer when listening to infant cry sounds and control sounds (i.e., scrambled sounds, see [12]), while they simultaneously view a picture of their own or an unknown child. The edited picture of the fathers’ own child, previously used in the fMRI task to examine neural responses, is also used in the present task.

A total of three cry sounds are recorded from three infants (2 males, 1 female). Cry sounds are recorded within the first two days after birth, using a TasCAM DR-05 solid-state recorder with a 44.a Khz sampling rate and 16 bit. All cry sounds are scaled, the intensity is normalized to the same mean intensity and sounds are edited using PRAAT software [36]. For each cry sound, a neutral control sound is created by calculating the average spectral density over the entire duration of the original sound. After this procedure, all cry sounds and control sounds are intensity matched. The control sounds are identical to the original auditory stimuli in terms of duration, intensity, spectral content, and amplitude envelope.

Participants are seated comfortably in front of a laptop screen wearing headphones, while holding the handgrip dynamometer in their dominant hand. During an unlimited practice period, participants are instructed to squeeze the handgrip dynamometer at full and half strength. Participants can see their performances being graphically displayed on a monitor. The monitor is directed away from the participant when they are able to modulate their handgrip strength. Participants have no insight into their performances during the experimental trials of the task.

The handgrip-force task is administered with a laptop using E-prime (Psychology Software Tools, Inc., Sharpsburg, PA, United States). Hand squeezes intensities (in kg) are transferred directly from the handgrip dynamometer to AcqKnowledge software (Biopac Systems, 2004). After the practice period, a baseline measure of handgrip strength is obtained. To prompt the participant, the words ‘squeeze maximally’ are displayed in the middle of the screen for 1 s, then a fixation is shown for 3 s, and subsequently the words ‘squeeze at half strength’ are shown for 1 s. Following this baseline measure, participants perform three trials requesting to squeeze at maximally and half strength in four randomly presented conditions [1]: viewing an image of their own infant while hearing scrambled control sounds [2]; viewing an image of their own infant while hearing cry sounds [3]; viewing an image of an unknown infant while hearing scrambled control sounds [4]; viewing an image of an unknown infant while hearing cry sounds. In each trial sounds and images are presented for 12 s. After 8 s participants are prompted to squeeze at maximum strength (instruction displayed for 1 s) followed by a request for half handgrip strength (instruction displayed for 1 s). A fixation cross is shown for 3 s between full- and half handgrip strength prompts.

In line with previous studies [12, 13], modulation of handgrip strength will be calculated by dividing half-strength squeeze intensity by full-strength squeeze intensity, so that scores above 0.5 indicate the use of excessive handgrip force when a half-strength squeeze is requested.

#### Early childhood experiences

To examine the moderating role of fathers’ early childhood experiences, fathers report on the Conflict Tactics Scale – Parent Child (CTS [39];), which measures experienced abuse and neglect during childhood. Participants also complete a questionnaire measuring use of parental love withdrawal, containing 11 items. Participants report on 7 items of the Love Withdrawal subscale of the Children’s Report of Parental Behavior Inventory (CRPBI [40, 41];), from which two items were slightly adapted for a better translation. Four items from the Parental Discipline Questionnaire (PDQ [42];) were added to obtain a more comprehensive measurement of parental love withdrawal. The 11-item questionnaire has been frequently used in previously research [11, 17, 43–45]. Reliability and validity of the CRPBI and its subscales had been established [41] and see also [46].

#### Background variables

We included measures on various background variables to control for confounding effects or to compare and combine the present dataset with other datasets collected as part of the Father Trials project. During each visit participants report on their quality of sleep, personal health and hygiene (developed for the purpose of this study), and the Positive and Negative Affect Schedule ((PANAS [47];). After the first visit, participants complete the following questionnaires at home: the Baby Care Questionnaire (BCQ [48];), Daily Life (DL; developed for the purpose of this study), Edinburgh Postnatal Depression Scale (EPDS [49];), The Family Assessment Device (FAD [50];), Gender Specific Orientation Questionnaire (GSOQ, developed for the purpose of this study), Highly Sensitive Person Scale (HSPS [51];), Parental Protection Questionnaire (PPQ; developed for the purpose of this study), Moral Identity Questionnaire (MIQ [52];), and the Task Division questionnaire (TD; developed for the purpose of this study).

After the first visit, online questionnaires are also sent to the partner of the participant. The partner completes the following questionnaires at home: BCQ (developed for the purpose of this study), FAD [50], DL (developed for the purpose of this study), EPDS [49] and TD (developed for the purpose of this study). The ethics committee of the Leiden University Medical Center (LUMC) approved all questionnaires. Questionnaires developed for the purpose of this study have been previously used in a pilot study.

Testosterone, estradiol and cortisol levels are measured to explore relations with oxytocin and vasopressin levels. Measures of testosterone, estradiol and cortisol are obtained prior to administration of oxytocin, vasopressin or placebo, 30 min after administration and at the end of the research visit (approximately 2 h after administration). Participants collect at least 1.5 ml saliva by expectorating down a straw into a test tube. Samples are immediately stored in a refrigerator at − 80 degrees Celsius until laboratory assessment.

Hair samples of approximately 3–5 mm (diameter) and minimally 3 cm length are cut to measure mean cortisol and testosterone levels of the past few months (see also [53]). Hair samples are cut around the inion of the occipital protuberance, as close to the scalp as possible. Hair samples are taped to a paper on which it is indicated which hair strands are closest to the scalp. Hair samples are placed into tin aluminum foil packages and stored at room temperature until laboratory assessment. Hair color and other potential confounders in the measurement of mean cortisol and testosterone levels are taken into account using a questionnaire (see “Confounders in the measurement of Corticosteroids in Hair (CoMCoH) [53]).

### Statistical analyses

To examine the effects of oxytocin and vasopressin administration on parenting behavior and the behavioral and neural responses to infant signals, statistical analyses will be performed within the general (ized) linear mixed model framework (GLMM). Using GLMM, we can account for the hierarchical structure of the data (i.e., repeated measurements nested within participants). Statistical analyses will be performed using appropriate statistical software (e.g., Statistical Package for the Social Sciences (SPSS), R or Mplus. Statistical analyses will be performed with an alpha level of .05 (corrected for multiple testing using appropriate methods, for example the Benjamini-Hochberg procedure [54]). To explore moderation by fathers’ early childhood experiences, fathers’ early childhood experiences will be added as a moderator in the proposed GLMM models.

### Data management and ethics

Data will be handled confidentially. Data will be stored in the local computers systems of the Vrije Universiteit Amsterdam. Data is protected in accordance with the University protocol. Personal information and data are treated and processed conform the European Union General Data Protection Regulation and the Dutch Act on Implementation of the General Data Protection Regulation. Data and hair and saliva samples are coded using a participant numbering system. Names and other information that can directly identify participants is omitted. Data cannot be traced back to participants in scientific reports and publications about the study. The researchers, the committee that monitors the safety of the study, the Medical Ethical Committee of the LUMC, and the Inspection for the Healthcare have access to the data. All persons with access to the data keep the data confidential. Participants who have any questions or complaints about the processing of personal information can contact the principal investigator of the present study, the Institutional Data Protection Officer, or de Dutch Protection Authority.

The research protocol (NL70143.058.19) has been approved by the ethics committee of the LUMC. Participants provide written consent for participation and the use and storage of data. Data and hair and saliva samples are stored for 15 years. Both parents provide written consent for participation of their child, and the use and storage of data concerning their child. Participants are informed that participation is voluntary and that they can withdraw from the study at any time, without providing any reason and without any consequences. Consent forms and all communication material have been approved by the ethics committee of the LUMC and are available upon request from the corresponding author. Participants can contact an independent expert who is available during the course of the study for questions and advice.

There are no risks associated with the assessments used in the study. Possible side effects of oxytocin are negligible [32, 33]. No adverse effects have been reported in participants undergoing fMRI at the currently used field strengths.

Participants will be informed about the study findings via newsletters, which are produced every six months. Furthermore, we will disseminate findings through peer-reviewed publications, scientific conferences, interviews, and public lectures. Any changes in the research protocol are communicated to the Netherlands Trial Registry (NTR), ethics committee of the LUMC and to BMC Psychology. When necessary, additional consent is obtained from participants. Authorships of publications will be based on recommendations for the Conduct, Reporting, Editing, and Publication of Scholarly Work in Medical Journals [55]. The trial is registered in the NTR (Trial ID: NL8124), Date registered: October 29, 2019).

## Discussion

The present study will examine the hormonal, behavioral, and neural underpinnings of paternal behavior in first-time fathers during a specific phase of fatherhood: between two and seven months after the baby has been born. The current study protocol presents a randomized, double-blind, placebo-controlled within-subject trial in which we aim to examine the effects of intranasal administration of oxytocin and vasopressin on parenting behavior and the neural and behavioral responses to infant signals. Moreover, we will examine brain-behavior associations, taking into account the effects of oxytocin and vasopressin. Finally, we will examine whether behavioral and neural effects are moderated by fathers’ early childhood experiences.

### Strengths and limitations

The experimental within-subject design of the study is the most important strength of the study. A randomized, double-blind, placebo-controlled within-subject design is considered the gold standard in testing intervention effects. Randomization ensures unbiased assignment of participants to the conditions and blind assessments eliminate (un) conscious human influence on research outcomes. Furthermore, it is the first study examining oxytocin and vasopressin administration in a within-subject design including first-time fathers in the early phase of fatherhood. Most previous research has focused on maternal caregiving, the outcomes of this study will provide important insights into the hormonal, behavioral and neural correlates of fathers’ parenting behavior and will contribute to a better and broader understanding about fatherhood. Another strength of the study is that effects of oxytocin and vasopressin administration are measured on a behavioral and neural level, which will contribute to an improved understanding of brain-behavior associations.

Limitations of the study should also be noted. First, we will recruit participants through municipality records. Fathers have to express their interest in participation with the attached response card, which may result in selection bias. However, by recruitment via municipality records all fathers in the general population are invited to participate in the study and random assignment of participants to all three conditions limits the potential influence of selection bias on our study outcomes. Another limitation is the inclusion of first-time fathers during a specific phase of fatherhood: when the infant is between two and seven months old. However, a focus on fathers in the early postnatal period is important as this marks a period in which men adapt and grow into their new role of being a father. Nevertheless, the results may not be generalizable to fathers with two or more children and to first-time fathers with children in an older age range.

## Data Availability

The datasets generated during and/or analyzed during the current study are available from the corresponding author on reasonable request.

## Abbreviations

AST: Auditory Startling Task
AVP: Vasopressin
fMRI: Functional magnetic resonance imaging
GLMM: General (ized) linear mixed model framework
ICC: Intercoder reliability
ISI: Inter stimulus interval
LUMC: Leiden University Medical Center
NTR: Netherlands Trial Registry
OXT: Oxytocin
SPSS: Statistical Package for the Social Sciences
